# Identification of Shearer Cutting Patterns Using Vibration Signals Based on a Least Squares Support Vector Machine with an Improved Fruit Fly Optimization Algorithm

**DOI:** 10.3390/s16010090

**Published:** 2016-01-12

**Authors:** Lei Si, Zhongbin Wang, Xinhua Liu, Chao Tan, Ze Liu, Jing Xu

**Affiliations:** 1 School of Mechatronic Engineering, China University of Mining & Technology, No. 1 Daxue Road, Xuzhou 221116, China; sileicool@163.com (L.S.); l_xinhua_2006@126.com (X.L.); tccadcumt@126.com (C.T.); cumt_liuze@163.com (Z.L.); xujingcumt@126.com (J.X.); 2 School of Information and Electrical Engineering, China University of Mining & Technology, No. 1 Daxue Road, Xuzhou 221116, China

**Keywords:** shearer cutting pattern identification, least squares support vector machine, fruit fly optimization algorithm, ensemble empirical mode decomposition, feature extraction

## Abstract

Shearers play an important role in fully mechanized coal mining face and accurately identifying their cutting pattern is very helpful for improving the automation level of shearers and ensuring the safety of coal mining. The least squares support vector machine (LSSVM) has been proven to offer strong potential in prediction and classification issues, particularly by employing an appropriate meta-heuristic algorithm to determine the values of its two parameters. However, these meta-heuristic algorithms have the drawbacks of being hard to understand and reaching the global optimal solution slowly. In this paper, an improved fly optimization algorithm (IFOA) to optimize the parameters of LSSVM was presented and the LSSVM coupled with IFOA (IFOA-LSSVM) was used to identify the shearer cutting pattern. The vibration acceleration signals of five cutting patterns were collected and the special state features were extracted based on the ensemble empirical mode decomposition (EEMD) and the kernel function. Some examples on the IFOA-LSSVM model were further presented and the results were compared with LSSVM, PSO-LSSVM, GA-LSSVM and FOA-LSSVM models in detail. The comparison results indicate that the proposed approach was feasible, efficient and outperformed the others. Finally, an industrial application example at the coal mining face was demonstrated to specify the effect of the proposed system.

## 1. Introduction

In a fully mechanized coal mining face, as the most important coal mining equipment, a shearer uses a drum to cut the coal. Due to the poor working conditions of coal mining, shearer operators does not have an accurate way to determine whether the shearer drum is cutting coal, rock, or coal with gangue depending only on simple visualization. This can lead to some poor coal quality and low mining efficiency problems. Moreover, in collieries many accidents are occurring with increasing frequently. The main reason of the problems is that the automation level of coal mining equipment is too low. With the development the suitable automation techniques, the automatic control of shearers has attracted more and more attention and accurate monitoring of shearer working status has played an indispensable important role for the automatic control of shearers. Therefore, researching the identification approach for shearer cutting patterns has become a challenging and significant subject [1].

Traditional identification techniques for shearer cutting patterns are mostly based on coal-rock recognition. The most influential methods are *γ*-ray detection [2], radar detection [3], infrared detection [4], and image detection [5], *etc.* However, these methods cannot satisfy the needs of practical applications and possess lower recognition rates because of the harsh conditions in practical production operation. In this context, this paper refers to the fault diagnosis and pattern recognition methods for traditional equipment and focuses on the identification method for shearer cutting patterns. Sensors are extensively used in pattern recognition and a diagnosis system to tackle the problem of perception by providing information about the machine. Using vibrations to collect the state information has become an effective method. In this regard, vibration-based analysis is becoming the most commonly used method and also proved to be efficient in various real applications. For a shearer, the rocker arm is the critical component and the vibrations of the rocker arm can comprehensively reflect the cutting conditions of the shearer, which can be diagnosed correctly by appropriate measurement and processing of sensor signals.

In general, existing pattern recognition methods can be classified into two categories: model-based methods and data-driven methods. The model-based pattern recognition aims to determine a pattern using the system’s analytical/mathematical model(s). However, the analytical/mathematical model associated a specific pattern is difficult to construct accurately, which leads to the ineffectiveness of model-based methods. In recent years, with the development of intelligent computing technology, data-driven methods have received much attention. In these methods, the pattern diagnosis can be realized by mapping the pattern space to the feature space through some modern intelligent algorithms [6], such as expert systems [7,8], neural networks [9,10], fuzzy logic [11], rough sets [12], and their hybrid methods [13,14]. Although the neural network and other conventional artificial intelligent techniques have been widely used in fault diagnosis and pattern recognition, they require sufficient samples and have limitations in generalization of results in models that can over-fit the samples because of the empirical risk minimization principle. Support vector machine (SVM) is a machine learning algorithm advocating structural risk minimization principle and has been widely used in classification and regression prediction because of its desirable generalization performance. The least squares support vector machine (LSSVM) is a reformulation of SVM which leads to solving a linear Karush-Kuhn-Tucker (KKT) system. The LSSVM can deal with non-linear systems and perform with high precision, making it a powerful tool for modeling and forecasting non-linear systems [15,16,17]. The performance of a LSSVM model largely depends on the values of its two parameters, one of which (denoted regularization parameter “*C*”) controls the tradeoff between margin maximization and error minimization. Another is called the kernel parameter and can implicitly define the nonlinear mapping from input space to high-dimensional feature space. Therefore, it is an indispensable step to optimize the parameters of LSSVM for a good performance in handling a learning task. Currently, several meta-heuristic algorithms have been employed to determine the appropriate values of these two parameters, such as particle swarm optimization [18,19], genetic algorithm [20,21], ant colony algorithm [22], and immune algorithm [23]. However, these optimization algorithms have the common drawbacks of being hard to understand and reaching the global optimal solution slowly.

The fruit fly optimization algorithm (FOA) proposed by Pan [24] is a novel evolutionary computation and optimization technique. This new optimization algorithm has the advantages of being easy to understand and to be written into program code which is not too long compared with other algorithms. More recently, FOAs have been applied in a variety of fields, such as power load forecasting [25], neural network parameter optimization [26], PID controller parameter tuning [27], design and optimization of key control characteristics [28], and so on. However, it often suffers the problem of being trapped into a local optimum which leads to premature convergence. In this research, an improved fruit fly optimization algorithm (IFOA) is proposed to optimize the two parameters of the LSSVM model, named the IFOA-LSSVM model, which uses a fruit fly optimization algorithm with two improvements to efficiently control the global search of LSSVM model in shearer cutting pattern identification.

The remaining parts of the paper are organized as follows: Section 2 summarizes some related works about the state of the art approaches to the problem. Section 3 introduces the basic theory of the original LSSVM and FOA methods, and presents the proposed IFOA-LSSVM model in detail. Section 4 describes the identification system of shearer cutting patterns based on the proposed method. Section 5 provides some examples and comparisons of IFOA-LSSVM with other methods. Section 6 presents the application results of the proposed method on a coal mining face. Section 7 gives the conclusions of our paper and proposes some future work.

## 2. Related Works on Identification of Shearer Cutting Patterns

In the past decades, many researchers have focused on coal-rock identification to roughly estimate the cutting state of shearers and many kinds of coal-rock recognition methods have been successively proposed. In [2], gamma-ray backscatter sensing was used to measure the boundary coal thickness. In [3], a radar coal thickness sensor was developed to identify the coal-rock interface and measure the thickness of a coal seam. In [5], a coal-rock interface identification method was provided based on the processing of visible light and infrared images. In [29], the recognition of a coal-rock interface in the top caving was investigated via the vibration signals of the tail beam of the hydraulic support. In [30], the color, grey scale, grain, shape and other image features of visible light images and infrared images were integrated and used to identify the coal-rock interface. In [31], the wavelet packet were utilized to extract the features of the torsional vibration signal of a drum shaft and the extracted features were integrated to recognize the coal-rock interface through fuzzy neural network technology. In [32], acoustic detection was applied in the identification of the coal-rock interface according to the sonic wave reflection and refraction at the water-coal interface and coal-rock interface. In [33], Sahoo *et al.* carried out some experiments about the application of a opto-tactile sensor for recognizing rock surfaces. In [34], the radar technology was used to identify the coal-rock interface and obtain the cutting patterns of a shearer. 

Although many coal-rock recognition methods have been developed, they have some common disadvantages. Firstly, the coal-rock detectors in the above references are complex and require too harsh coal seam geological conditions, which cannot satisfy extensive applications during practical production. Furthermore, the recognition rate is sensitively influenced by the conditions of gangue included in the coal seam. Therefore, this paper utilizes the data-driven theory and proposes an intelligent identification method for shearer cutting patterns based on the integration of least squares support vector machine and an improved fruit fly optimization algorithm.

## 3. Least Squares Support Vector Machine with Improved Fruit Fly Optimization Algorithm

### 3.1. Least Squares Support Vector Machine

The support vector machine, a reliable tool for solving pattern recognition and classification problems, was initially presented by Vapnik and his coworkers in 1995 based on statistical learning theory and the structural risk minimization principle [35]. The least squares support vector machine (LSSVM) is an extension of SVM which applies the linear least squares criteria to the loss function instead of inequality constraints [36].

In a set of samples ${\left\{x_{i},y_{i}\right\}}_{i=1}^{m}$, $x_{i}\in R^{n}$ is the input data and $y_{i}\in R^{n}$ is the corresponding output value for sample *i*. The formulation of the primal problem for the LSSVM can be given as follows:

(1) $$\text{min }\underset{w,b,\xi }{J}\left(w,\xi \right)=\frac{1}{2}w^{T}w+\frac{1}{2}C\sum_{i=1}^{m}\xi_{i}^{2}$$

subject to the equality constraint:

(2) $$y_{i}=w^{T}\varphi (x_{i})+b+\xi_{i},\;i=1,2,\cdots ,m$$

where *J* is objective function; 1/2*w^T^w* is used as a flatness measurement function; *C* is the regularization parameter, which determines the tradeoff between the training error and the model flatness; *ξ_i_* is the slack variable; the nonlinear mapping φ maps the input data into a high dimensional feature space, where a linear regression problem is obtained and solved; *b* is the bias, and *w* is a weight vector of the same dimension as the feature space.

The Lagrangian function *L* can be constructed by:

(3) $$L\left(w,b,\xi ,\alpha \right)=\frac{1}{2}w^{T}w+\frac{1}{2}C\sum_{i=1}^{m}\xi_{i}^{2}-\sum_{i=1}^{m}\alpha_{i}\left[w^{T}\varphi (x_{i})+b+\xi_{i}-y_{i}\right]$$

where *α_i_* is Lagrange multiplier. The Karush-Kuhn-Tucker (KKT) conditions for optimality are given by:

(4) $$\left\{ \begin{array}{l} \frac{\partial L}{\partial w}=0\Rightarrow w=\sum_{i=1}^{m}\alpha_{i}\varphi (x_{i})\\ \frac{\partial L}{\partial b}=0\Rightarrow \sum_{i=1}^{m}\alpha_{i}=0\\ \frac{\partial L}{\partial \xi_{i}}=0\Rightarrow \alpha_{i}=C\xi_{i}\\ \frac{\partial L}{\partial \alpha_{i}}=0\Rightarrow w^{T}\varphi (x_{i})+b+\xi_{i}-y_{i}=0 \end{array}\right.$$

Based on Equation (3), one can formulate a linear system *Ax* = *B* in order to represent this problem as:

(5) $$\left[\begin{array}{l} 0\;I^{T}\\ I\;K+C^{-1}I \end{array}\right]\left[\begin{array}{l} b\\ A \end{array}\right]=\left[\begin{array}{l} 0\\ Y \end{array}\right]$$

where *I* = [1, 1, …, 1]^T^, *A* = [*α*_1_, *α*_2_, …, *α_m_*]^T^, *Y* = [*y*_1_, *y*_2_, …, *y_m_*]^T^. According to the Mercer’s condition, the Kernel function can be set as:

(6) $$K(x_{i},x_{j})=\varphi {\left(x_{i}\right)}^{T}\cdot \varphi (x_{j})$$

Then, the regression function of LSSVM model can be described as follows:

(7) $$f(x)=\sum_{i=1}^{m}\alpha_{i}K(x,x_{i})+b$$

For a classification problem, *y*_i_ ∈ {−1, 1} indicates the corresponding desired output vector and the classification decision function is described as follows:

(8) $$f(x)=\text{sgn}\left(w^{T}\varphi (x)+b\right)$$

Equation (1) for classification problem should meet the following equality constraint:

(9) $$y_{i}\left[w^{T}\varphi (x_{i})+b\right]=1-\xi_{i},\;i=1,2,\cdots ,m$$

The corresponding optimization problem of LS-SVM model with Lagrange function is described as follows:

(10) $$L\left(w,b,\xi ,\alpha \right)=\frac{1}{2}w^{T}w+\frac{1}{2}C\sum_{i=1}^{m}\xi_{i}^{2}-\sum_{i=1}^{m}\alpha_{i}\left\{y_{i}\left[w^{T}\varphi (x_{i})+b\right]-1+\xi_{i}\right\}$$

Using the same processing method, the classification decision function is described as follows:

(11) $$f(x)=\text{sgn}\left(\sum_{i=1}^{m}\alpha_{i}y_{i}K(x,x_{i})+b\right)$$

There are several different types of Mercer kernel function *K*(*x*, *x_i_*) such as sigmoid, polynomial and radial basis function (RBF). The RBF is a common option for the kernel function because of fewer parameters that need to be set and an excellent overall performance [37]. Therefore, this paper selected the RBF as the kernel function:

(12) $$K(x,x_{i})=\text{exp}(-\frac{1}{2\delta^{2}}{\left\|x-x_{i}\right\|}^{2})$$

Consequently, there are two parameters that need to be chosen in the LSSVM model, which are the bandwidth of the Gaussian RBF kernel “*δ*” and the regularization parameter “*C*”. Many researches have shown that the LSSVM parameters have great influence on its learning and generalization ability. This paper presents an improved fruit fly optimization algorithm to determine the optimal values of these two parameters, so that LSSVM could perform the best generalization ability.

### 3.2. The Basic FOA and Analysis

**Figure 1:**
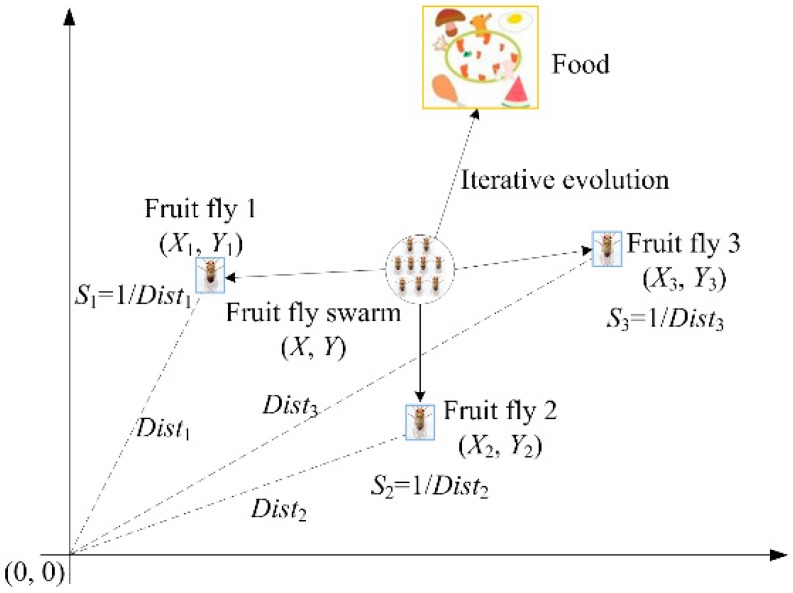
Food searching iterative process of fruit fly swarm.

The fruit fly optimization algorithm (FOA) is a new swarm intelligence algorithm, which was proposed by Pan [30], and it is a kind of interactive evolutionary computation method. The basic thought of FOA is that fruit fly finds the food through the food finding behavior. During finding food, a fruit fly initially smells a particular odor by using its osphresis organs, sends and receives information from its neighbors and compares the current best location and fitness. Flies identify the fitness values by taste and fly toward the location with better fitness. They use their sensitive vision to seek food and fly toward that direction further. Figure 1 shows the food finding iterative process of a fruit fly swarm.

According to the food finding characteristics of fruit fly swarm, the FOA can be divided into several steps, as follows:

*Step 1: Parameters initialization*. The swarm location range (*LR*), maximum iteration number (*Maxgen*), and population size (*sizepop*) are initialized. The initial fruit fly swarm location (*X_axis*, *Y_axis*) and the random flight distance range *FR* should be initialized first:

(13) $$\begin{array}{l} X\_axis=\text{rand}(LR)\\ Y\_axis=\text{rand}(LR) \end{array}$$

*Step 2: Population initialization*. The random direction and distance for food searching of any individual fruit fly can be given as follows:

(14) $$\begin{array}{l} X_{i}=X\_axis+\text{rand}(FR)\\ Y_{i}=Y\_axis+\text{rand}(FR) \end{array}$$

*Step 3: Population evaluation*. Firstly, the distance of food source to the initialization location (*Dist_i_*) is calculated by using the following equation:

(15) $$Dist_{i}=\sqrt{X_{i}^{2}+Y_{i}^{2}}$$

Secondly, the smell concentration judgment value (*S_i_*) need to be calculated, and the value of *S_i_* is the reciprocal of the distance *Dist_i_*:

(16) $$S_{i}=\frac{1}{Dist_{i}}$$

Then, the smell concentration (*Smell_i_*) of the individual fruit fly location is calculated by inputting the smell concentration judgment value (*S_i_*) into the *Smell_i_* judgment function (also called the fitness function). Finally, the fruit fly with minimum smell concentration (the minimal value of *Smell_i_*) among the swarm is determined and found out:

(17) $$\begin{array}{l} Smell_{i}=Function(S_{i})\\ \left[bestSmell\;bestIndex]=\text{min}(Smell\right) \end{array}$$

*Step 4: Vision searching process*. The minimal concentration value and *X*, *Y* coordinate are maintained. The fruit fly swarm flies toward the location with the minimal smell concentration value by using vision:

(18) $$\begin{array}{l} Smellbest=bestSmell\\ X\_axis=X(bestIndex)\\ Y\_axis=Y(bestIndex) \end{array}$$

*Step 5: The iterative optimization is entered to repeat the implementation of Steps (2)–(4).* When the smell concentration reaches the preset precision value or the iterative number reaches the maximal *Maxgen*, the circulation stops. Through the analysis of Equations (14)–(15), it can be found that FOA has some disadvantages which limit its searching performance. The disadvantages are summarized below.

(1) It is clear that the value *S_i_* is non-negative and this smell concentration judgment value is then substituted into the smell concentration judgment function to find the smell concentration of the individual location of the fruit fly. That is to say that the variable of the fitness function is in the zone of (0, +∞), which will prevent the application of FOA in some problems with negative numbers in the domain.
(2) FOA depends only on the current generation optimal solution and when the optimal individual is found, all fruit flies will fly towards this individual. Then the fruit flies are updated according to Equation (10). This operation will greatly reduce the diversity and exploration ability of fly swarm. Furthermore, the current generation optimal solution may not the global optimum and inapposite *FR* will make the FOA get into the local optimal solution.

### 3.3. The Improved Strategies for FOA

Based on the aforementioned analysis, the original FOA has demanding application conditions and can possibly get into a local extreme. Thus, two improvements for the original FOA are proposed in this subsection.

(1) In Step (3), in order to ensure the variable of the fitness function is in the zone of (−∞, +∞), the smell concentration judgment value (*S_i_*) can be calculated by the following equation:
  (19) $$S_{i}=\frac{\text{sign}[2\times \text{rand}(\;)-1]}{Dist_{i}}$$
(2) In order to improve the diversity and exploration ability of fly swarm and increase the ability to break away from the local optimum, this paper proposes an improved strategy for FOA through expanding search in the initial phase and narrowing search in the later phase. Let the fruit fly population be updated by the following equation:
  (20) $$\begin{array}{l} X_{i}=X\_axis+\text{rand}(FR)\times \beta^{\frac{gen}{\eta }}\\ Y_{i}=Y\_axis+\text{rand}(FR)\times \beta^{\frac{gen}{\eta }} \end{array}$$
  where *β* is defined as the adjustment factor; *η* is used to control the flight distance range *FR* and can be determined according to the practical problem; *gen* is the current number of iterations. In the first phase, the random flight distance range should increase to realize the diversity of population. The adjustment factor *β* should be larger than 1, marked as *β*_1_ and the number of iterations in this phase *t*_1_ is equal to *gen*. Thus, the fruit fly population can be updated as follows:
  (21) $$\begin{array}{l} X_{i}=X\_axis+[(b-a)\times \text{rand}(\;)+a]\times \beta_{1}^{\frac{t_{1}}{\eta }}\\ Y_{i}=Y\_axis+[(b-a)\times \text{rand}(\;)+a]\times \beta_{1}^{\frac{t_{1}}{\eta }} \end{array}$$
  where [*a*, *b*] denotes the flight distance range of fruit fly. In the second phase, the random flight distance range should increase to enhance the convergence accuracy and convergence speed. The adjustment factor *β* should be smaller than 1, marked as *β*_2_ and the number of iterations in this phase *t*_2_ is equal to *gen* − *t*_1_. Thus, the fruit fly population can be updated as follows:
  (22) $$\begin{array}{l} X_{i}=X\_axis+[(b-a)\times \text{rand}(\;)+a]\times \beta_{2}^{\frac{t_{2}}{\eta }}\\ Y_{i}=Y\_axis+[(b-a)\times \text{rand}(\;)+a]\times \beta_{2}^{\frac{t_{2}}{\eta }} \end{array}$$

**Figure 2:**
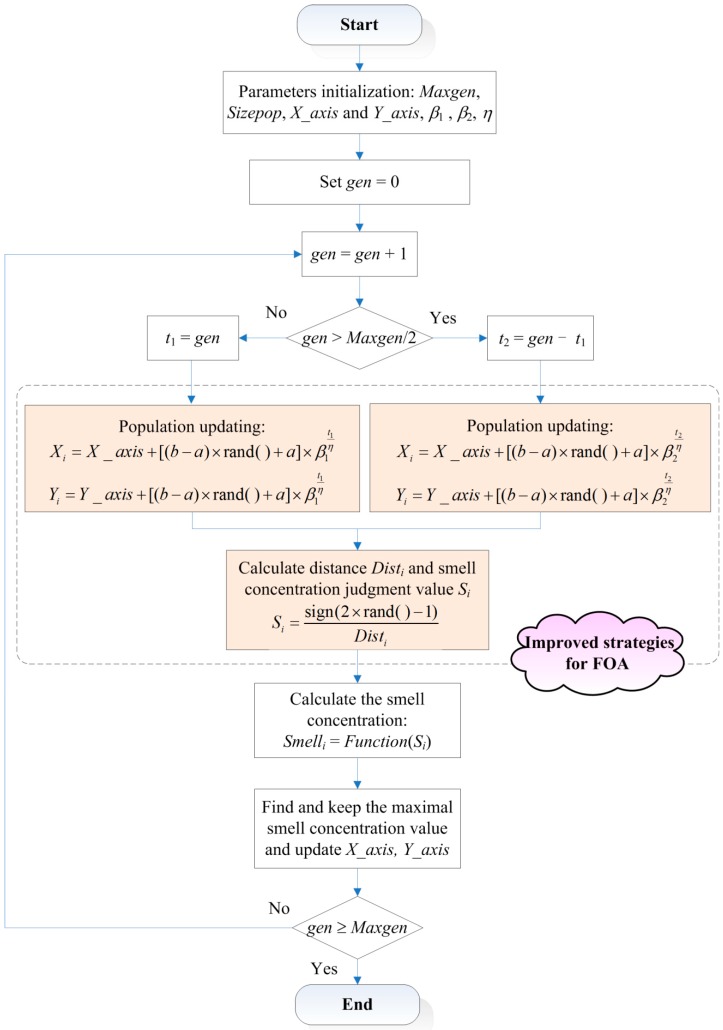
The flowchart of improved FOA.

The complete flowchart of improved FOA is shown in Figure 2.

### 3.4. Improved Fruit Fly Optimization Algorithm for Parameters Selection of LSSVM Model

Selecting appropriate bandwidth “δ” and regularization parameter “C” of LSSVM is extremely important for the classification performance of LSSVM. In this paper, the proposed IFOA is used to choose the appropriate parameter values of the LSSVM model, named IFOA-LSSVM model. The details of IFOA for parameters determination of the LSSVM model are as follows:

*Step 1: Initialization parameters*. The maximum iteration number Maxgen, the population size sizepop, the initial fruit fly swarm location (X_axis, Y_axis), and the flight distance range FR should be determined at first. In the LSSVM model, two parameters need to be determined and we can set X_axis = rands(1, 2), Y_axis = rands(1, 2), where rands( ) denotes the random number generation function. Set gen = 0.

*Step 2: Evolution starting*. In the IFOA-LSSVM program, we employ two variables [*X*(*i*, :), *Y*(*i*, :)] to represent the flight distance for food finding of an individual fruit fly *i*. If *gen* ≤ *Maxgen*/2, the flight direction of fruit fly *i* should be updated by Equation (21). If *gen* > *Maxgen*/2, the flight direction of fruit fly *i* should be updated by Equation (22).

*Step 3: Calculation*. In the IFOA-LSSVM program, *D*(*i*,1) and *D*(*i*,2) are used to represent the distance *Dist_i_* of the fruit fly *i* to the origin, which can be calculated as follows:

(23) $$D(i,1)=\sqrt{X{\left(i,1\right)}^{2}+Y{\left(i,1\right)}^{2}},D(i,2)=\sqrt{X{\left(i,2\right)}^{2}+Y{\left(i,2\right)}^{2}}$$

Similarly, we can use *S*(*i*, 1) and *S*(*i*, 2) to describe the smell concentration judgment value *S_i_* and it can be calculated as follows:

(24) $$S(i,1)=\frac{\text{sign}[2\times \text{rand}(\;)-1]}{D(i,1)},S(i,2)=\frac{\text{sign}[2\times \text{rand}(\;)-1]}{D(i,2)}$$

In the proposed model, the parameters (*C*, *δ*) of LSSVM are represented by *S*(*i*, 1) and *S*(*i*, 2), and can be set as *C* = 20× |*S*(*i*, 1)|, *δ* = |*S*(*i*, 2)|, respectively. Then, the smell concentration *Smell_i_* (also called the fitness value of fruit fly *i*) should be calculated. We adopt two fitness functions to represent the regression prediction performance and classification ability of IFOA-LSSVM model. One is the root-mean-square error (*RMSE*) between the outputs of LSSVM and actual values and another is the classification error rate (*CER*).

*Step 4: Updating*. The fruit flies are operated according to Equations (19) and (20), and then the swarm is updated through Equations (21) and (22). The smell concentration values are calculated again. Set *gen* = *gen* +1.

*Step 5: Iteration termination*. When *gen* reaches the max iterative number, the termination criterion satisfies, and the optimal parameters (C*, δ*) of LSSVM model can be obtained. Otherwise, go back to Step 2.

## 4. The Identification System for Shearer Cutting Pattern Based on Proposed Method

**Figure 3:**
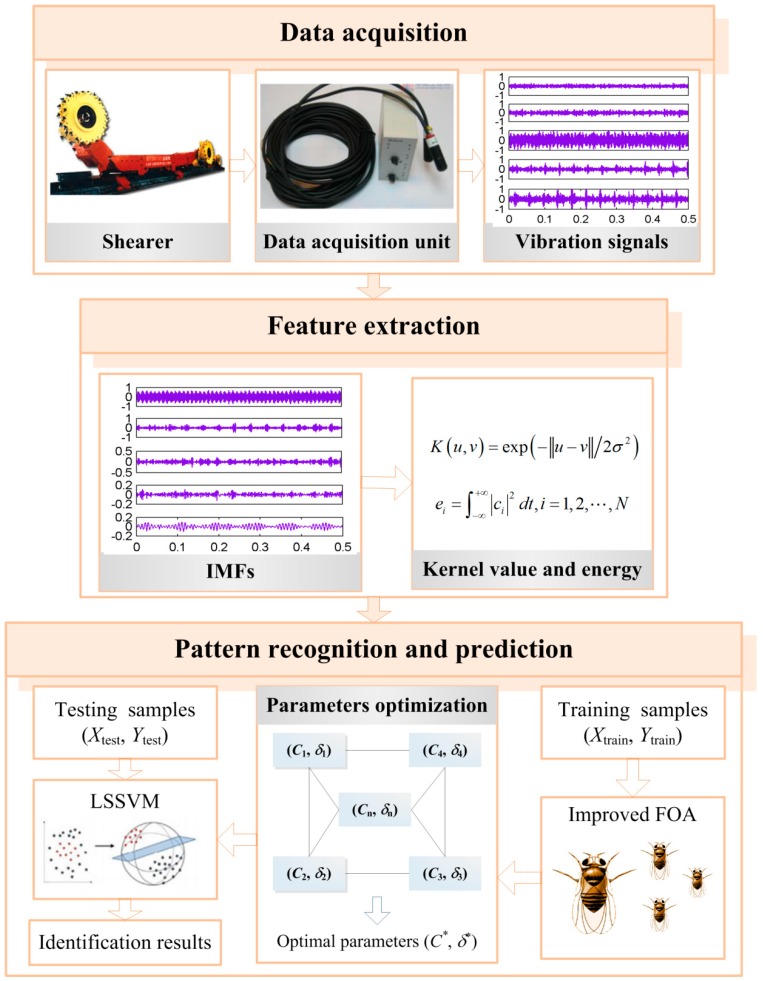
The identification system for shearer cutting pattern based on proposed method.

The intelligent identification for shearer cutting pattern based on proposed method is essentially a pattern recognition system, shown in Figure 3. It mainly consists of data acquisition, feature extraction and pattern recognition and prediction, which is explained as follows.

### 4.1. Data Acquisition

**Figure 4:**
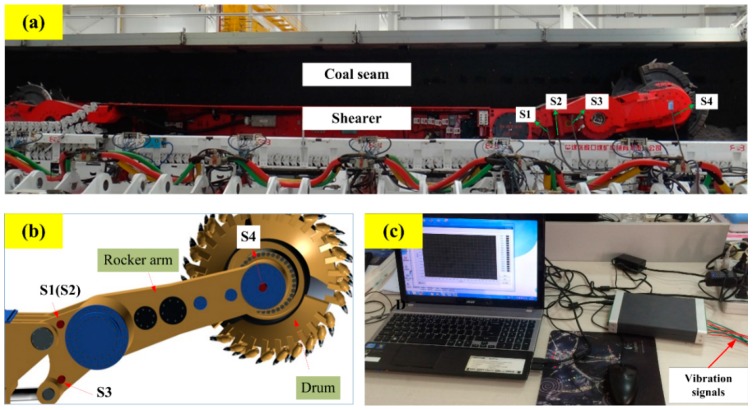
Self-designed experimental system for shearer cutting coal: (**a**) The experiment bench of shearer cutting coal; (**b**) The installation sketch of accelerometers; (**c**) Vibration signals processing device.

**Figure 5:**
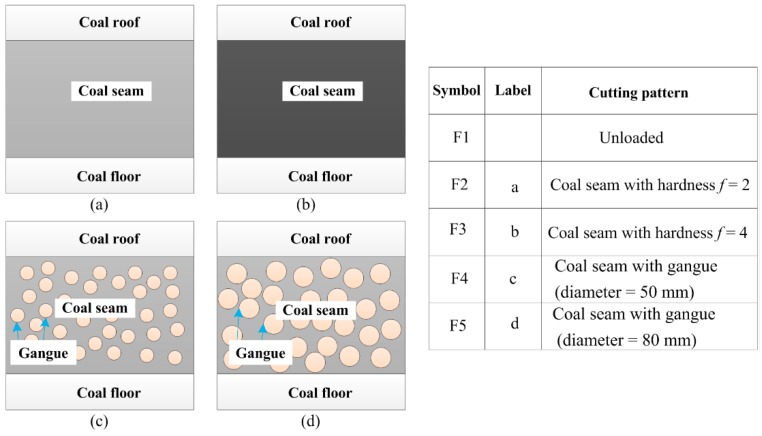
Different geological conditions of coal seam.

The cutting pattern diagnosis of shearers starts with data acquisition to collect the machinery working information. Vibration signal acquisition is the most commonly used method which is realized by sensors. In this study, the data were acquired through four sensors installed in a self-designed experimental system for a shearer cutting coal, as shown in Figure 4. In the experiment, the coal seam was mainly divided into four parts, including two kinds of coal seams with different hardness and the coal seam with some strata of gangue. All cutting patterns of the shearer (including the shearer with unloaded condition) are represented in Figure 5.

**Figure 6:**
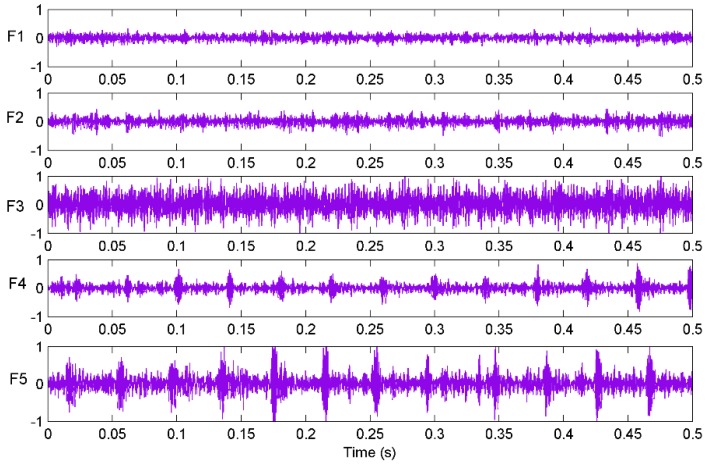
Vibration signals from sensor S1 in different cutting patterns.

In Figure 4, the signs “S1, S2, S3 and S4” refer to four accelerometers located on the rocker arm. A multifunctional high-speed collector performed the data acquisition and the data were collected into a notebook computer through the USB interface. The sampling frequency was set as 12 kHz and the sampling time of each sample was 0.5 s. Vibration signals of sensor S1 with different patterns are plotted in Figure 6. Finally, 400 groups of samples were obtained with 80 groups of samples for each cutting pattern.

### 4.2. Feature Extraction

The signal feature extraction is a critical initial step in any pattern recognition and fault diagnosis system. The extraction accuracy has a great influence on the final identification results, so there have been a lot of signal processing approaches to obtain desirable features for machinery pattern diagnosis, among which the Fast Fourier Transform (FFT) and Wavelet Transform (WT) are widely used and well-established. When a fault occurs, new frequency components may appear and a change of the convergence of the frequency spectrum may take place. However, for weak signals the features are submerged in the strong background noise and it is difficult to extract effective features by traditional feature extraction methods. Fortunately, the ensemble empirical mode decomposition (EEMD) has been proposed in [38]. The EEMD method adds a certain amount of Gaussian white noise in the original signal before decomposing it, so as to solve the problem of frequency aliasing. This method is very appropriate for non-stationary and non-linear signals [39,40]. The steps of EEMD can be briefly summarized as follows:

(1) Determine the number of ensemble *M* and initialize the amplitude of the added white noise, and set *m* = 1.
(2) Add a white noise series with the given amplitude to the original signal.
  (25) $$x_{m}(t)=x(t)+a_{m}(t)$$
  where *a_m_*(*t*) denotes the *m*th added white noise series and *x_m_*(*t*) denotes the investigated signal added white noise (noise-added signal) of the *m*th trial.
(3) By the use of EMD method [41], the noise-added signal *x_m_*(*t*) is decomposed into *N* intrinsic mode functions (IMFs), which can be marked as *b_nm_*(*t*)(*n*=1,2,…,*N*) and *b_nm_*(*t*) represents the *n*th IMF of the *m*th trial.
(4) If *m* < *M* then let *m* = *m* + 1. Repeat Steps (2) and (3) again with different white noise series each time until *m* = *M*.
(5) Calculate the ensemble mean *b_n_*(*t*) of the *M* trials for each IMF. Then output the mean *c_i_*(*t*) (*i* = 1,2,…,*N*) of each of the *N* IMFs as the final decomposed results:
  (26) $$c_{i}(t)=\frac{1}{M}\sum_{m=1}^{M}b_{n,m}(t),n=1,2,\cdots ,N$$

**Figure 7:**
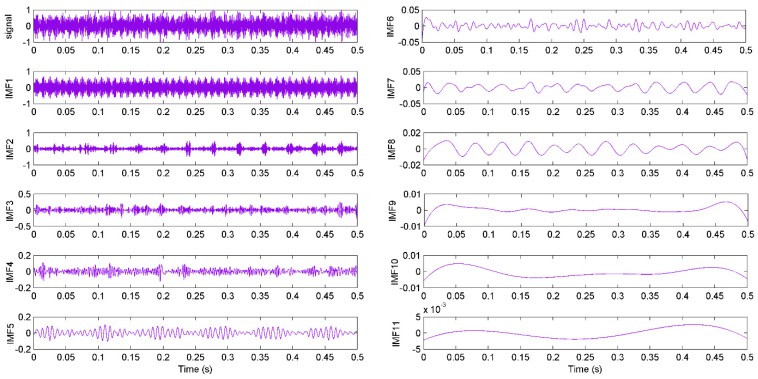
The decomposed components with EEMD and original signal from S1 at F3.

According to the above steps, a measured signal is decomposed and the decomposition results are given in Figure 7. It shows 11 IMFs in different frequency bands decomposed by the EEMD algorithm. It can be seen from the figure that the original signal is very complicated and the decomposed IMFs are hard to use for state diagnosis. Hence, features of the signals need to be extracted. In addition, the correlation coefficients between the last three IMFs (IMF9, IMF10 and IMF11) and the original signal are too low. Therefore, the kernel function value and energy of the first eight IMFs were extracted and used as features for pattern identification [42].

The kernel feature is employed by a kernel function. Firstly, the signal collected by the *i*th sensor is defined as a sequence $x_{i}=\{x_{i1},x_{i2},\cdots ,x_{il}\}$, where *i* = 1, 2, …, *S* and *S* is the number of sensors, *l* is the number of sampling points. Then the sequence is decomposed by EEMD to get *N* IMFs: {*c*_1_,*c*_2_,…,*c_N_*}, where *c_k_* = {*c_k_*_1_,*c_k_*_2_,…,*c_kl_*}, *k* = 1,2,…,*N*.

The 2-norm of the *k*th IMF can be calculated as follows:

(27) $$norm_{k}={\left\|c_{k}\right\|}_{2}=\sqrt{\sum_{j=1}^{j=l}c_{kj}^{2}}$$

Then, a vector can be constructed from *N* IMFs:

(28) $$NORM=\left\{norm_{1},norm_{2},\cdots ,norm_{N}\right\}$$

A Gaussian kernel function is described as $K\left(u,v\right)=\text{exp}\left(-\left\|u-v\right\|/2\sigma^{2}\right)$. If *v* is defined as a vector {0}_1__×*N*_, the kernel feature value of the signal from the *i*th sensor can be calculated as *kf_i_* = *K*(*NORM, v*). Finally, a kernel feature sample *KF* can be obtained by calculating *S* sensors’ signal data:

(29) $$KF={\left\{kf_{1},kf_{2},\cdots ,kf_{S}\right\}}^{T}$$

We assume that *e_i_* is the energy of the *i*th IMF, which can be calculated as follows:

(30) $$e_{i}=\int_{-\infty }^{+\infty }{\left|c_{i}\right|}^{2}dt,i=1,2,\cdots ,N$$

The maximum energy of the IMFs is used to generate the energy sample *EF*, shown as follows:

(31) $$EF=\left\{e_{\text{max}1},e_{\text{max}2},\cdots ,e_{\text{max}S}\right\}$$

In this experiment, the parameter of the Gaussian kernel function *σ* was set as 5. A feature sample could be constructed by the *KF*, *EF* or the combination of *KF* and *EF*. The performance of the proposed model based on different features was investigated and analyzed in Section 5.

### 4.3. Pattern Recognition and Prediction

In the shearer cutting pattern identification system, support vector machine, especially the least squares support vector machine, is widely used as a pattern recognition and prediction approach to diagnose which kind of working pattern the machinery is in. The proposed fruit fly optimization algorithm is adopted to determine the optimal parameters in a least squares support vector machine.

## 5. Example Computation and Comparison Analysis

**Figure 8:**
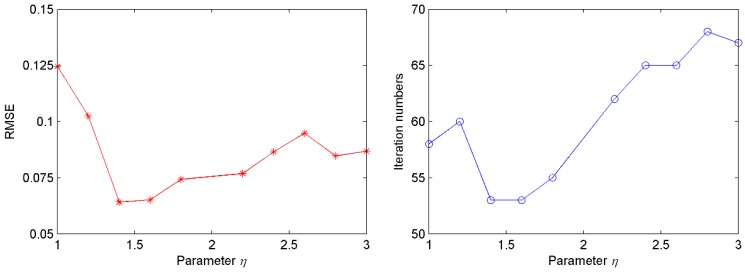
The influence of different *η* on the performance of proposed method.

In this example, all samples were collected from the self-designed experimental system shown in Figure 4. Four hundred groups of samples were obtained with 80 groups of samples for each cutting pattern. The corresponding cutting patterns could be quantized as 1, 2, 3, 4 and 5. Seventy five percent of the samples were used as the training samples to optimize the parameters of LSSVM and the remaining samples were used for testing the generalization ability of the proposed model. The parameters were provided as follows: *Maxgen* = 100, *sizepop* = 20, (*X_axis,Y_axis*) ⊂ [−1, 1], *FR* = [−10, 10] (thus *a* = −10, *b* = 10), *β*_1_ = 1.1, *β*_2_ = 0.9. The parameter *η* has great influence on the quality of solution and searching speed. For different *η*, the changes of training root-mean-square error (*RMSE*) and iteration numbers to obtain the *RMSE* are shown in Figure 8. Seen from this figure, the proposed method has better training *RMSE* and iteration numbers when the parameter *η* is equal to 1.4, so the value of parameter *η* is set as 1.4 in this experiment.

In order to measure the prediction performance of an IFOA-LSSVM model, the classification error rate (*CER*) and the difference between the output of the model and the desired output were considered as the evaluation indexes and represented in separate ways. In this paper, the following measures were employed for model evaluation: the *RMSE*, the mean absolute error (*MAE*), the mean relative error (*MRE*), and Theil’s inequality coefficient (*TIC*). The *CER* represented the categorization performance of IFOA-LSSVM. The *RMSE*, *MAE* and *MRE* confirmed the prediction accuracy of the proposed model. The *TIC* indicated the level of agreement between the proposed mode land the studied process. These indicators were defined as follows:

(32) $$CER=\frac{\text{number of wrongly identifed samples}}{n}$$

(33) $$RMSE=\sqrt{\frac{\sum_{i=1}^{n}{\left(y_{i}-{\hat{y}}_{i}\right)}^{2}}{n}}$$

(34) $$MAE=\frac{1}{n}\sum_{i=1}^{n}\left|y_{i}-{\hat{y}}_{i}\right|$$

(35) $$MRE=\frac{1}{n}\sum_{i=1}^{n}\frac{\left|y_{i}-{\hat{y}}_{i}\right|}{y_{i}}\times 100\%$$

(36) $$TIC=\sqrt{\frac{\frac{1}{n}\sum_{i=1}^{n}{\left(y_{i}-{\hat{y}}_{i}\right)}^{2}}{\sqrt{\sum_{i=1}^{n}{y_{i}}^{2}+\sum_{i=1}^{n}{{\hat{y}}_{i}}^{2}}}}$$

where *n* is the number of samples; $y_{i}$ denotes the actual value of the *i*th sample; ${\hat{y}}_{i}$ denotes the LSSVM output of the *i*th sample.

As described in the feature extraction subsection, our feature construction included two types of features: kernel feature (*KF*) and energy feature (*EF*). Here we constructed three models based on *KF*, *EF*, the combination of *KF* and *EF* (*KF* + *EF*), respectively. In order to reduce the random error, the models were trained and tested for about 50 times based on different features and the average values were computed to compare. Finally, the performance of IFOA-LSSVM model trained and tested with various features was shown in Table 1.

**Table 1:** The performance of IFOA-LSSVM on the testing set with different combinations of features.

Training Features	*CER*	*RMSE*	*MAE*	*MRE* (%)	*TIC*
*KF*	0.1782	0.3291	0.1754	7.79	0.0669
*EF*	0.2193	0.4262	0.2014	9.83	0.0939
*KF* + *EF*	0.03015	0.0611	0.0524	2.66	0.0089

According to the results in Table 1, the performance of proposed model trained with the combination of *KF* and *EF* showed obvious improvement as compared to the individual feature. This demonstrated that both of the two types of features contributed to identifying the cutting patterns of shearer. Henceforth, the combination of *KF* and *EF* was selected as the feature samples to learn the proposed model.

In order to investigate the performance of proposed IFOA-LSSVM model, four other models: FOA-LSSVM, PSO-LSSVM (LSSVM optimized by particle swarm optimization algorithm), GA-LSSVM (LSSVM optimized by genetic algorithm), and single LSSVM were employed for comparison. The parameters of PSO were set as: population size = 20, maximum iteration number = 100, acceleration factors C_1_ = 1.5 and C_1_ = 1.7. The parameters of GA were set as: population size = 20, maximum iteration number = 100, crossover probability = 0.5, mutation probability = 0.1. The configurations of experimental environment for these methods were uniform. The models were trained and tested about 50 times and the average values were computed to compare. The comparison results of different models on the testing samples are listed in Table 2.

**Table 2:** Comparison of LSSVM, PSO-LSSVM, GA-LSSVM, FOA-LSSVM and IFOA-LSSVM models.

Model	Optimal Parameters		*CER*	*RMSE*	*MAE*	*MRE* (%)	*TIC*
	C*	δ*					
LSSVM	10	2	0.1096	0.2609	0.1401	6.17	0.0544
PSO-LSSVM	32.5671	3.8547	0.08108	0.1304	0.0815	4.11	0.0238
GA-LSSVM	15.1136	1.2256	0.06915	0.1107	0.0831	3.71	0.0289
FOA-LSSVM	19.3742	0.2827	0.05011	0.0694	0.0645	2.88	0.0112
IFOA-LSSVM	28.3846	0.0513	0.03015	0.0611	0.0524	2.66	0.0089

In Table 2, the optimal parameters (C* and δ*) of the five models denote the parameters with the smallest *CER* of different models among the 50 testing results. According to the results of IFOA tuning the parameters of the LSSVM model, the optimal values of *C* and *δ* were selected as 28.3846 and 0.0513, respectively. In the FOA-LSSVM model, the optimal values of *C* and *δ* were 19.3742 and 0.2827. According to the results of GA and PSO optimizing the parameters of LSSVM model, the values of *C* and *δ* were optimized as 15.1136 and 1.2256, 32.5671 and 3.8547, respectively. In the single LSSVM model, the values of *C* and *δ* were chosen as 10 and 2. The specific error indexes listed in Table 2 indicate that the IFOA-LSSVM model performed better than other models. In details, the LSSVM model provided a *CER* of 0.1096, a *RMSE* of 0.2609, a *MAE* of 0.1401, a *MRE* of 6.17%, and a *TIC* of 0.0544. The PSO-LSSVM model obtained a *CER* of 0.08108, a *RMSE* of 0.1304, a *MAE* of 0.0815, a *MRE* of 4.11%, and a *TIC* of 0.0238. The GA-LSSVM model produced a *CER* of 0.06915, a *RMSE* of 0.1107, a *MAE* of 0.0831, a *MRE* of 3.71%, and a *TIC* of 0.0289. The FOA-LSSVM model obtained a *CER* of 0.05011, a *RMSE* of 0.0694, a *MAE* of 0.0645, a *MRE* of 2.88%, and a *TIC* of 0.0112. Finally, the IFOA-LSSVM model could acquire a *CER* of 0.03015, a *RMSE* of 0.0611, a *MAE* of 0.0524, a *MRE* of 2.66%, and a *TIC* of 0.0089. We could see that the performances of LSSVM coupled with other optimization algorithms were much better than single LSSVM. One noticed that selecting the parameters in LSSVM model was of considerable significance for improving the learning and generalization performance of LSSVM. As shown in Table 2, the IFOA-LSSVM gave a better performance than PSO-LSSVM, GA-LSSVM and FOA-LSSVM models. Our proposed IFOA-LSSVM was reliable to provide superior regression prediction performance and classification ability for shearer cutting pattern.

**Figure 9:**
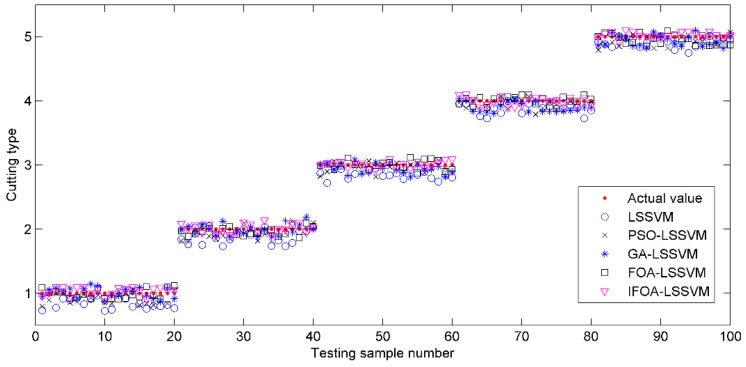
The prediction values of testing samples based on different models.

**Figure 10:**
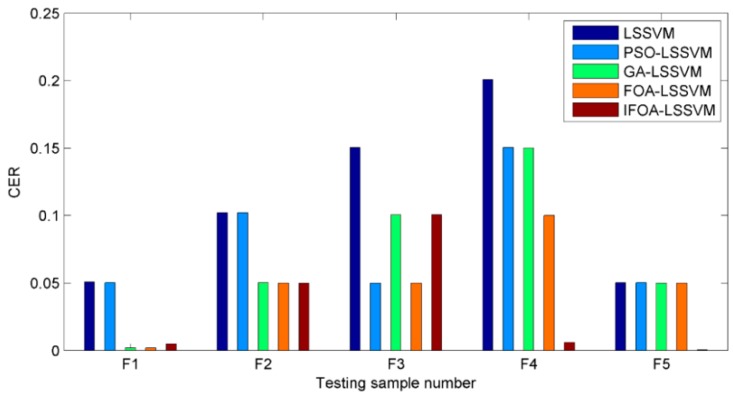
The *CER*s of five cutting types based on different models.

Visual comparison illustrations of the prediction values and classification results are also shown in Figure 9 and Figure 10. Obviously, the prediction results of IFOA-LSSVM model were nearly the real values on most data points, and the proposed model’s classification performance was superior to that of the competing models.

**Figure 11:**
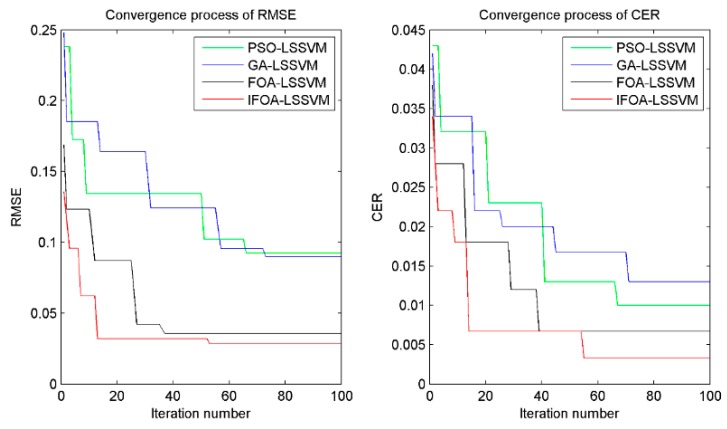
Comparison of PSO, GA, FOA and IFOA for optimization process.

In order to investigate the efficiency of the proposed method, the convergence performance of four models (PSO-LSSVM, GA-LSSVM, FOA-LSSVM and IFOA-LSSVM) was compared and analyzed. The convergence curves of *RMSE* and *CER* obtained through the four different algorithms are illustrated in Figure 11. The results indicate that the proposed method had the advantage of faster convergence to the global optimal fitness by about 20 iteration numbers than PSO-LSSVM and GA-LSSVM. Although the iteration numbers were a little larger than that of FOA-LSSVM, the final *RMSE* and *CER* were excellent. Besides, the *RMSE* and *CER* by IFOA-LSSVM were 0.0285 and 0.0033, while the values by PSO-LSSVM, GA-LSSVM and FOA-LSSVM were 0.0924 and 0.010, 0.0898 and 0.013, 0.0355 and 0.0067, respectively, which signified the proposed method performed higher accuracy for forecasting and classification than other three methods. In briefly, the computation results manifested that the proposed IFOA-LSSVM model had better performance in the efficiency and generalization ability of identifying shearer cutting pattern.

## 6. Industrial Application

**Figure 12:**
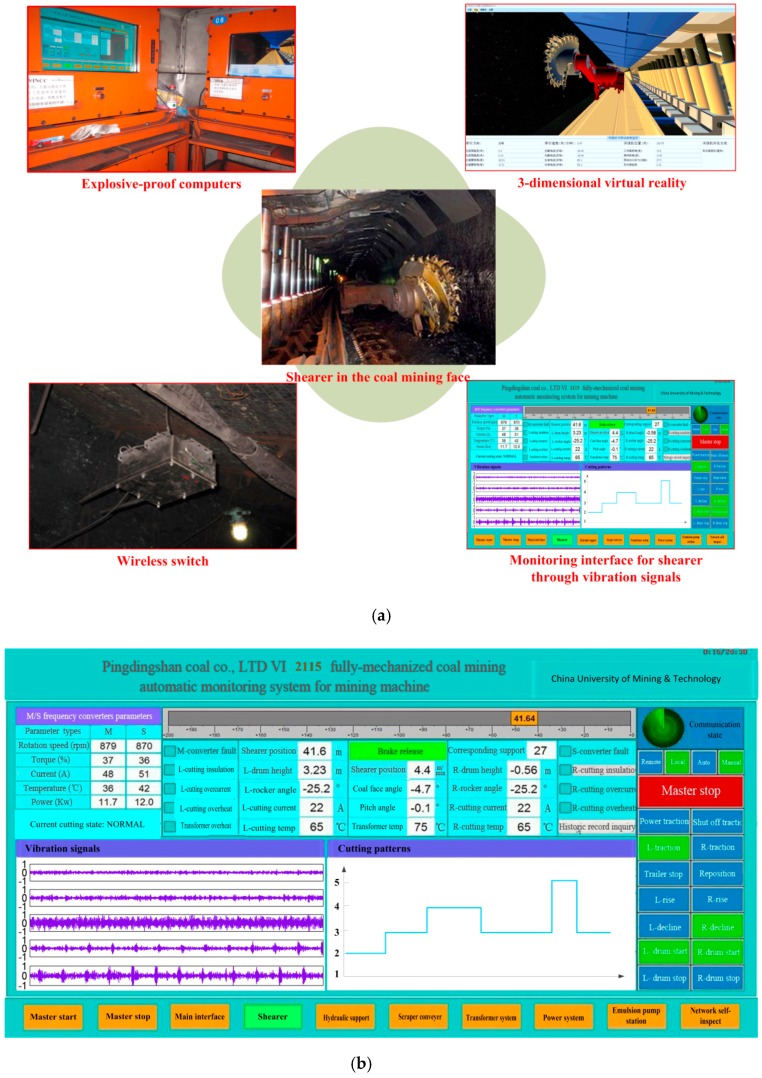
Basic structure of the system for industrial test in the coal mining face: (**a**) The system in coal mining face based on proposed method; (**b**) The monitoring interface for shearer.

In order to verify the application possibilities of the proposed shearer control method, a system based on the proposed method has been developed and an industrial test was carried out on a coal mining face. The basic structure of the system is shown in Figure 12. The application was accomplished at the 2115 coal mining face in the No.13 Mine of the Pingdingshan Coal Industrial Group Corporation. Seen from this figure, the accelerometers were installed inside of the shearer rocker arm shell to guarantee the reliability. The vibration signals were collected and transmitted into the explosion-proof computers by wireless switches. The computers could process the signals and execute the proposed method to identify the shearer cutting pattern. Meanwhile, a 3-dimensional virtual reality system was used to vividly display the working status of the shearer.

**Figure 13:**
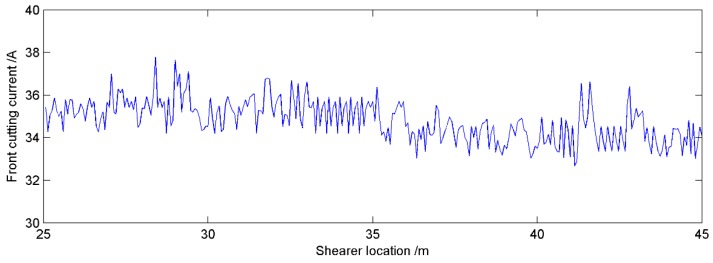
Front cutting current curve according to proposed method.

The goal of the proposed method is to accurately identify the cutting pattern of the shearer, which can provide the basis for its automatic control. Therefore, the cutting current of the front cutting motor was plotted in Figure 13 when the shearer was working from 25 m to 45 m. In this monitoring interval, the front cutting current was changed in the scope of 32.6894 A to 37.7769 A and the average value was 34.8074 A. The maximum current was only about 8.53% larger than that of the average value. The results indicated that the shearer could work smoothly and safely in the coal seam according to the identification provided by the proposed method and the system was proved stable and reliable in the practical application.

## 7. Conclusions and Future Work

In this paper, we propose a novel method for identifying the shearer cutting pattern based on least squares support vector machine optimized by improved fruit fly optimization algorithm (IFOA-LSSVM). This proposed method uses the IFOA to automatically select the appropriate parameters of the LSSVM model in order to improve the forecasting and classification accuracy. The training features are constructed reasonably by the combination of kernel feature and energy feature. To validate the proposed method, four other alternative models (single LSSVM, PSO-LSSVM, GA-LSSVM, and FOA-LSSVM) are employed to compare the forecasting and classification performances. Example computation results show that the *CER*, *RMSE*, *MAE* *MRE* and *TIC* of proposed model are much smaller than those obtained by the competing models. Meanwhile, the convergence speed and precision of IFOA-LSSVM model perform with significant superiority over other alternative models in terms of the shearer cutting pattern identification. Furthermore, the industrial application result indicates that the system based on proposed method can provide stable and reliable references for the automatic control of a shearer.

In future studies, the authors will analyze the vibrations of other parts to represent the influence on the results of classification and plan to investigate advanced feature extraction methods to further improve the pattern identification results. Possible improvements may include some intelligent algorithms for synchronous feature selection and parameter optimization to obtain better performance. In addition, applications of the proposed method in the fault diagnosis domain are also worth further study.
